# 1752. Impact of a multifaceted, outpatient antimicrobial stewardship intervention bundle on unnecessary antimicrobial prescribing in upper respiratory tract infections (URI)

**DOI:** 10.1093/ofid/ofac492.1382

**Published:** 2022-12-15

**Authors:** Ryan W Stevens, Paschalis Vergidis, Darrin Christopherson, Evan Draper, Benjamin J Anderson, Laura Dinnes, Nipunie S Rajapakse, Harry R Powers, Kevin Epps, Kellie Arensman Hannan, Sara Ausman, Christina G Rivera, Sarah R Lessard, Kimberly Prigge, Abinash Virk, Kelsey L Jensen

**Affiliations:** Mayo Clinic, Rochester, Minnesota; Mayo Clinic, Rochester, Minnesota; Mayo Clinic, Rochester, Minnesota; Mayo Clinic, Rochester, Minnesota; Mayo Clinic, Rochester, Minnesota; Mayo Clinic, Rochester, Minnesota; Mayo Clinic, Rochester, Minnesota; Mayo Clinic, Rochester, Minnesota; Mayo Clinic, Rochester, Minnesota; Mayo Clinic Health System, Mankato, Minnesota; Mayo Clinic Health System - Eau Claire, Eau Claire, Wisconsin; Mayo Clinic, Rochester, Minnesota; Mayo Clinic Health System La Crosse WI, La Crosse, Wisconsin; Mayo Clinic, Rochester, Minnesota; Mayo Clinic, Rochester, Minnesota; Mayo Clinic Health System - Southeast Minnesota, Osage, Iowa

## Abstract

**Background:**

URIs are the most common indication for outpatient antibiotic prescribing. Given high rates of unnecessary prescribing, these indications have been identified as a high-priority target for outpatient antimicrobial stewardship programs (ASP). Our primary objective was to evaluate the impact of a system-wide, multifaceted, outpatient ASP intervention bundle on unnecessary antibiotic prescribing for URI.

**Methods:**

This quasi-experimental study was conducted from 2019 to 2021. ICD-10 codes for URIs were grouped into 3 tiers (i.e., tier I = antibiotics always indicated, tier II = sometimes, tier III = never). Encounters from 5 care specialties (i.e., family medicine, community internal medicine, express care, pediatrics, and emergency department) with a tier III URI primary ICD-10 code but without a secondary tier I or tier II code were included. COVID-19 ICD-10 codes were excluded. Interventions included construction of a prescribing data model, dissemination of clinician prescribing data and education, promotion of symptom management strategies, a patient-facing commitment poster, and a pre-populated URI order panel. Tools were designed at a system level and implemented by regional champions beginning in the 3rd quarter of 2020. The primary outcome was the rate of antibiotic prescribing, and the secondary outcome and counterbalance measure was the rate of repeat URI-related healthcare contact within 14 days. Outcomes were analyzed with chi-square with an α level of 0.05.

**Results:**

A total of 147403 encounters were included. The overall antibiotic prescribing rate decreased from 24.1% to 12.3% between 2019 and 2021 (p< 0.01). Significant reductions in tier III antibiotic prescribing were demonstrated for each region, care specialty, and syndrome evaluated (Table 1). A reduction in repeat healthcare contact was seen across the total cohort (9.5% in 2019 vs. 8.3% in 2021, p< 0.01); decreases in repeat contact rates were observed in those not initially receiving an antibiotic (10.3% vs. 8.6%, p< 0.01), but not in those who initially received an antibiotic (6.8% vs. 6.8%, p = 0.94).

Tier III URI encounter level antimicrobial prescribing rates by region, care specialty, and syndrome

**Figure:**
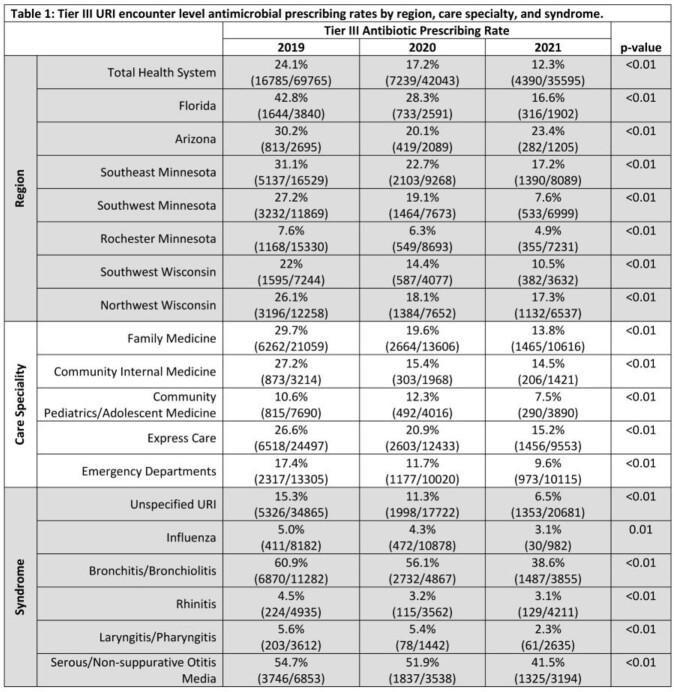

**Conclusion:**

A multifaceted, outpatient ASP intervention bundle decreased rates of unnecessary antimicrobial prescribing without increasing rates of 14-day repeat URI-related healthcare contact.

**Disclosures:**

**Paschalis Vergidis, MD**, AbbVie: DSMB|Cidara: Grant/Research Support|Scynexis: Grant/Research Support **Evan Draper, PharmD**, Gilead Foundation: Grant/Research Support **Sara Ausman, PharmD**, Gilead: Honoraria **Christina G. Rivera, PharmD**, Gilead: Grant/Research Support|Gilead: Honoraria|Insmed: Honoraria.

